# Whole-Genome SNP Association in the Horse: Identification of a Deletion in Myosin Va Responsible for Lavender Foal Syndrome

**DOI:** 10.1371/journal.pgen.1000909

**Published:** 2010-04-15

**Authors:** Samantha A. Brooks, Nicole Gabreski, Donald Miller, Abra Brisbin, Helen E. Brown, Cassandra Streeter, Jason Mezey, Deborah Cook, Douglas F. Antczak

**Affiliations:** 1 Department of Animal Science, College of Agriculture and Life Sciences, Cornell University, Ithaca, New York, United States of America; 2 Baker Institute for Animal Health, College of Veterinary Medicine, Cornell University, Ithaca, New York, United States of America; 3 Department of Biological Statistics and Computational Biology, College of Agriculture and Life Sciences, Cornell University, Ithaca, New York, United States of America; 4 M. H. Gluck Equine Research Center, Department of Veterinary Science, University of Kentucky, Lexington, Kentucky, United States of America; Stanford University School of Medicine, United States of America

## Abstract

Lavender Foal Syndrome (LFS) is a lethal inherited disease of horses with a suspected autosomal recessive mode of inheritance. LFS has been primarily diagnosed in a subgroup of the Arabian breed, the Egyptian Arabian horse. The condition is characterized by multiple neurological abnormalities and a dilute coat color. Candidate genes based on comparative phenotypes in mice and humans include the ras-associated protein RAB27a (*RAB27A*) and myosin Va (*MYO5A*). Here we report mapping of the locus responsible for LFS using a small set of 36 horses segregating for LFS. These horses were genotyped using a newly available single nucleotide polymorphism (SNP) chip containing 56,402 discriminatory elements. The whole genome scan identified an associated region containing these two functional candidate genes. Exon sequencing of the *MYO5A* gene from an affected foal revealed a single base deletion in exon 30 that changes the reading frame and introduces a premature stop codon. A PCR–based Restriction Fragment Length Polymorphism (PCR–RFLP) assay was designed and used to investigate the frequency of the mutant gene. All affected horses tested were homozygous for this mutation. Heterozygous carriers were detected in high frequency in families segregating for this trait, and the frequency of carriers in unrelated Egyptian Arabians was 10.3%. The mapping and discovery of the LFS mutation represents the first successful use of whole-genome SNP scanning in the horse for any trait. The RFLP assay can be used to assist breeders in avoiding carrier-to-carrier matings and thus in preventing the birth of affected foals.

## Author Summary

Genetic disorders affect many domesticated species, including the horse. In this study we have focused on Lavender Foal Syndrome, a seizure disorder that leads to suffering and death in foals soon after birth. A recessively inherited disorder, its occurrence is often unpredictable and difficult for horse breeders to avoid without a diagnostic test for carrier status. The recent completion of the horse genome sequence has provided new tools for mapping traits with unprecedented resolution and power. We have applied one such tool, the Equine SNP50 genotyping chip, to a small sample set from horses affected with Lavender Foal Syndrome. A single genetic location associated with the disorder was rapidly identified using this approach. Subsequent sequencing of functional candidate genes in this location revealed a single base deletion that likely causes Lavender Foal Syndrome. From a practical standpoint, this discovery and the development of a diagnostic test for the LFS allele provides a valuable new tool for breeders seeking to avoid the disease in their foal crop. However, this work also illustrates the utility of whole-genome association studies in the horse.

## Introduction

**Figure 1:**
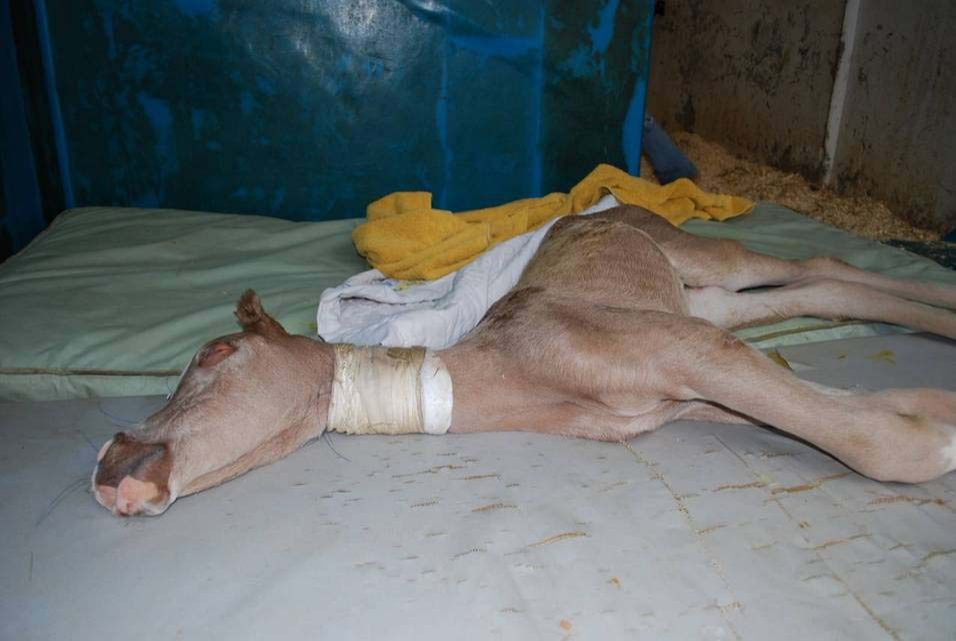
A foal with Lavender Foal Syndrome demonstrating opisthotonus, one cardinal neurological sign of the disorder. (Photo courtesy of Dr. Yael Giora).

Heritable disorders affect many domestic species, including the horse. In the Arabian breed of horse a neurological disorder has been reported that is lethal soon after birth [1]. Affected foals can display an array of neurological signs including tetanic-like seizures, opisthotonus, stiff or paddling leg movements and nystagmus (Figure 1) [2]. Mild leucopenia is sometimes observed [2], [3]. These neurologic impairments prevent the foal from standing and nursing normally and, if not lethal on their own, are often cause for euthanasia. In addition to these abnormalities, affected foals possess a characteristic diluted “lavender” coat color. This resulting coat color, variously described as pale gray, pewter, and light chestnut, as well as lavender, has coined the name “Lavender Foal Syndrome” (LFS) [2]. Also called “Coat Color Dilution Lethal” [2], there is currently no treatment for LFS available. Additionally, initial diagnosis can be difficult as the clinical signs of LFS can easily be confused with a number of neonatal conditions including neonatal maladjustment syndrome and encephalitis [2]. The inheritance of Lavender Foal Syndrome is suspected to be recessive, although extensive pedigree analysis has not, to date, been published. Outwardly healthy horses can sire lethally affected foals; therefore a recessive mode of inheritance for LFS is most likely.

Historically developed by the Bedouin tribesman on the Arabian Peninsula, the Arabian horse is one of the oldest recognized breeds of horse. Valued for its beauty and athleticism, the Arabian has contributed to the development of many light horse breeds, most notably the Thoroughbred, a breed used extensively in horse racing across the world [4]. The majority of documented cases of Lavender Foal Syndrome have been reported in the Egyptian Arabian, a sub-group of the Arabian breed found originally in Egypt but extensively exported and popular in the United States. Egyptian Arabians have their own registry, although they are also part of the main Arabian studbook. It is estimated that there are 49,000 living registered Egyptian Arabians worldwide (personal communication, Beth Minnich, Pyramid Society). Identifying the genetic basis of this condition and developing a diagnostic test for the LFS allele will enable breeders to make more informed selection of mating pairs, thus avoiding the production of affected foals and potentially lowering the frequency of this allele in the population, without wholesale culling of valuable stock.

Over the past 15 years the Horse Genome Project has produced several generations of analytical and diagnostic resources (genetic tools) that permit interrogation of polymorphisms across the entire equine genome [5], [6]. Previous mapping efforts using ∼300 microsatellite markers yielded results for several heritable diseases (for examples see [7], [8]). However, this small number of markers limited genetic studies in the horse to simple traits in closely related families with fairly large numbers of samples. The recently completed 6.8x whole genome sequence of the horse and the associated identification of approximately 1.5 million Single Nucleotide Polymorphisms (SNPs) located throughout the horse genomic sequence [9] has enabled the construction of a 56,402 element SNP chip for rapid whole genome scanning (Equine SNP50, Illumina, San Diego, CA). SNP-based whole genome association studies have proven exceptionally successful when studying simple mendelian traits in domesticated species. Two notable examples can be found in studies of coat traits in the dog [10] and recessive diseases of cattle [11].

Previously described mutations in mice and humans provide several comparative phenotypes similar to Lavender Foal Syndrome. Two genes in particular, Ras-associated protein RAB27a (*RAB27A*) and myosin Va (*MYO5A*), yield phenotypes with striking parallels to LFS. These two proteins, along with melanophilin (*MLPH*) are part of a transportation complex responsible for the trafficking of melanosomes to the periphery of the cell where they are transferred to the keratinocyte (reviewed in [12]). The myosin Va transport complex is also utilized in the dendrite of the neuron where it has been shown to move various cargo, including mRNAs, glutamate receptors, and secretory granules [13], [14]. Disruption of these diverse functions could explain the constellation of defects observed in *RAB27A* and *MYO5A* mutants. In mice, 71 mutations in *MYO5A* and 106 in *RAB27A* have been recorded in the MGD database [15]. In humans, several unique recessive mutations in these two genes have been shown to cause similar disorders. The severity of the phenotype, known as Griscelli syndrome, varies with the gene and location of the mutation [16]. Griscelli syndromes have been divided in to three categories based on the gene responsible; *MYO5A* in type 1, *RAB27A* in type 2, and *MLPH* in type 3 [17]. There are subtle differences in the phenotype of each of these subtypes. For example, *RAB27A* mutations in both human and mouse disrupt granule exocytosis in T lymphocytes. This leads to immunodeficiency and leukocyte infiltration in to vital organs, including the brain. Thus, although neurological defects are often present in *RAB27A* mutants they are usually secondary to this infiltration [17]. In contrast, *MYO5A* mutants exhibit a primary neurologic dysfunction and have normal immune function. Based on this distinction *MYO5A* was chosen as the primary candidate gene for Lavender Foal Syndrome.

## Results

### Pedigree Analysis

Pedigree data from the six affected foals available at the time of genotyping supported a recessive mode of inheritance. A single common ancestor was identified six to eight generations from these six affected foals (Figure S1). This common ancestor is present on both sides of the pedigree in each foal. This stallion may represent a founder among this group and this convergence in the pedigree supports identity by descent for the LFS mutation. Average inbreeding (F_i_) was 0.0861 for affected foals, versus 0.0394 for parents of foals. The extended pedigree also allowed for the calculation of the coancestry coefficient between each living relative and the nearest affected foal in the pedigree. Based on this calculation we predicted that the frequency of the LFS allele would be 0.42 among the 30 relatives used for genotyping.

### Association Mapping

**Figure 2:**
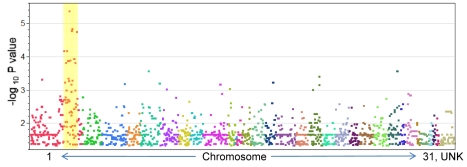
P-values (<0.05) for SNP association with Lavender Foal Syndrome. Individual chromosomes are represented with various colors in numerical order. The region with the most significant P-values (highlighted with a yellow bar) falls on the p arm of ECA1.

**Figure 3:**
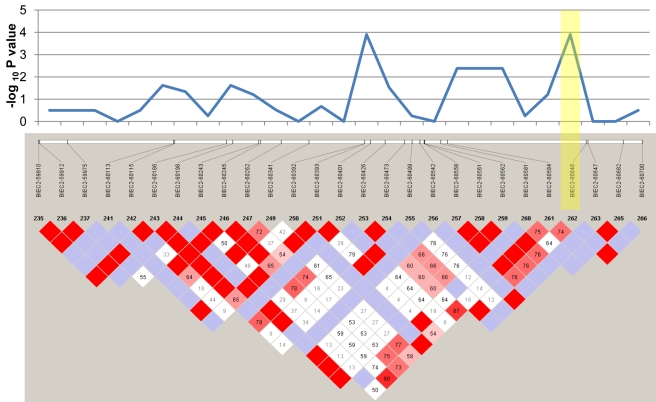
Linkage disequilibrium plot of the homozygous region associated with LFS. Plotted at the top are the corresponding p-values for these 27 SNPs. The yellow shaded box highlights the SNP closest to *MYO5A*. Red diamonds represent D' values equal to 1, lower values of D' are given in within the boxes in shades of pink to white, while non-significant associations are blue.

Genotypic association tests using the six affected foals and their 30 healthy relatives revealed a single region on chromosome 1 (ECA1) with statistical significance above that of the rest of the genome (Figure 2). These 14 highly significant SNPs encompassed a region spanning 10.5 Mb (ECA1:129228091 to 139718117). Although extensive inbreeding and relatedness between affected individuals produced a high number of coincidentally significant (p<0.05) SNPs across the genome, the high peak significance of SNPs in the candidate region (p = 4.62e-6) was convincing evidence for association. In total there were 14 SNPs at this locus that were more significantly associated with the LFS trait than any other region in the genome. The twelve LFS bearing chromosomes from the six affected horses represented only four unique haplotypes for this 10 Mb candidate region. These four haplotypes possessed one large block of 27 SNPs in common. This 1.6 Mb region was homozygous in all six affected horses and heterozygous in obligate carriers as well as many of the living relatives, as was predicted by the coancestry in the pedigree. The linkage disequilibrium (LD) structure and p-values in this likely location for a recessive mutation are plotted in Figure 3. Only 10 Ensembl Gene Predictions fell within this region, including *MYO5A*, but not *RAB27A* (UCSC Genome Browser [9]).

### Population Structure

Genome-wide observed homozygosity from the genotypes obtained using the EquineSNP50 chip was on average 65.14%. This was much higher than expected considering the homozygosity of the inbred mare chosen for whole genome sequencing was estimated at only 46% [9]. The ten founder Egyptian Arabian individuals from this study, as well as an additional 10 unrelated individuals from the Thoroughbred, Arabian (non-Egyptian) and Saddlebred breeds were used to calculate average genome-wide LD (Figure S2). This calculation revealed that the length of LD in the Egyptian was similar to that of the Thoroughbred, a breed with a long history of a closed studbook and relatively small foundation population. LD in the Egyptian was also much longer than that of the Arabian population as a whole, which was most similar to the Saddlebred. The Saddlebred breed registry was closed in 1917 and derived from fairly diverse types of horse suitable for use as transportation under saddle and in harness.

### Candidate Gene Sequencing

**Figure 4:**
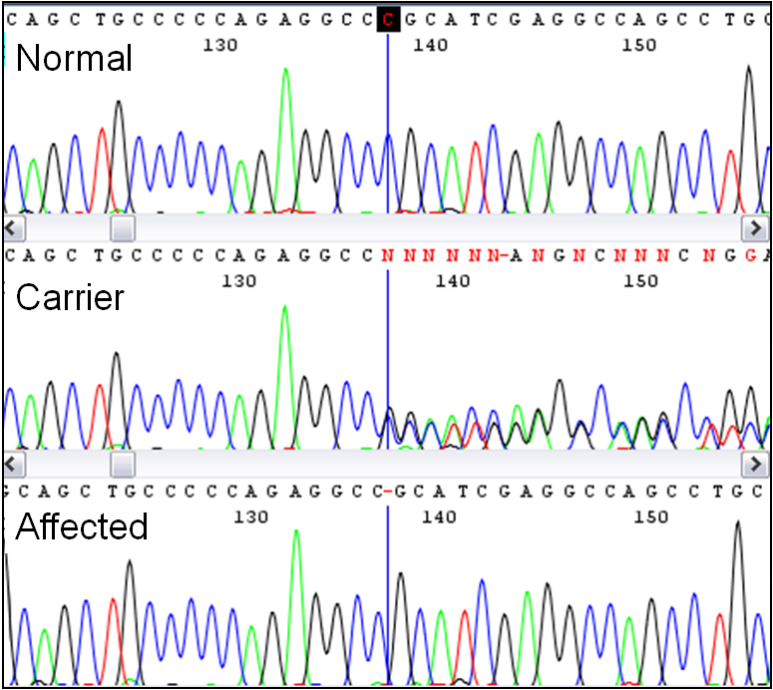
Discovery of the Lavender Foal Syndrome–associated single base deletion by sequencing. Pictured are aligned chromatograms from horses with each of the three genotypes. The deletion occurs at the base marked with the vertical line, causing a mixed sequence from this point forward in the carrier.

Individual PCR amplification and sequencing of the 39 exons of *MYO5A* from a LFS affected foal revealed three SNPs and one polymorphic microsatellite in intronic sequence, as well as a single base deletion in exon 30 of *MYO5A* (Table 1). This deletion was further confirmed by sequencing in a second foal and its heterozygous parents (Figure 4). The deletion is termed ECA1 g.138235715del per Human Genome Variation Society (http://www.hgvs.org/mutnomen/) nomenclature. This deletion changes the reading frame, creating a premature stop codon in the translation of exon 30, 12 amino acids following the mutation. A multiple alignment of the predicted LFS exon 30 amino acid sequence, as well as the wild type sequence from eight species, shows that this region of the myosin Va protein is highly conserved (Figure S3). The four intronic polymorphisms were not predicted to change the function of myosin Va and were therefore not investigated further.

**Table 1:** Polymorphisms identified in the *MYO5A* sequence.

HGVS Nomenclature	Within Gene Location	Type
g.138148824A>G	Intron 3+100	SNP
g.138168098G>A	Intron 6+189	SNP
g.138230294C>T	Intron 27–156	SNP
g.138235715del^a^	Exon 30+148	Frameshift
g.138253441GT(10_12)	Intron 35–79	(GT) satellite

(a- LFS associated polymorphism).

### Association and Frequency Estimates

We designed a PCR-RFLP assay using the Fau I restriction enzyme to detect this deletion (Figure S4). Digestion of the PCR product produces a positive control fragment of 289 bp in all genotypes. Presence of the deletion abolishes a Fau I site, changing the normal pattern of a 386 bp and a 90 bp fragment in to a single 476 bp product. All seven affected foals (the six originally submitted for mapping plus one additional obtained after mapping was completed) were homozygous for the deletion (Table 2). Eight out of the 14 parents of these affected foals were available for sampling and all carried the deletion. Among 23 relatives of affected foals 16 were identified as carriers of the deletion. A sample group of 114 Arabian horses was tested to provide a rough estimate of the frequency of the *MYO5A* exon 30 deletion, and therefore Lavender Foal Syndrome, in the breed as a whole (Table 3). 10.3% of Egyptian Arabians (six out of 58 horses) and 1.8% of non-Egyptian Arabians (one out of 56 horses) were identified as carriers.

**Table 2:** Association of Lavender Foal Syndrome with ECA1 g.138235715del.

Genotypes^a^	+/+	+/−	−/−
Affected (Homozygotes)	0	0	7
Parents (Carriers)	0	8	0
Other Relatives (Unknown Genotype)	7	16	0
Total	7	24	7

(a- “+” stands for the wild type while “−” indicates the deletion).

**Table 3:** Frequency of the Lavender Foal Syndrome associated deletion among a sample of Arabian horses.

Genotypes^a^	+/+	+/−	−/−	LFS Allele Freq.
Egyptian Arabian	52	6	0	0.052
Other Arabian	55	1	0	0.0089
Total	107	7	0	0.031

(a- “+” stands for the wild type while “−” indicates the deletion.

## Discussion

Here we describe the first successful use of the EquineSNP50 genotyping platform in identification of the mutation responsible for a genetic disorder in the horse. We have described a frameshift mutation in the *MYO5A* gene that leads to Lavender Foal Syndrome in the Egyptian Arabian breed of horse. This task was made more challenging by the small number (six) of DNA samples from available affected foals. We improved our chances of success by using pedigree data to select control samples from the extended family and by utilizing a genotype association rather than allelic association statistic in combination with identification of regions of homozygosity.

The extreme predicted impact on function resulting from the single base deletion in *MYO5A* exon 30 makes it a very logical cause of LFS. Indeed, an alignment of *MYO5A* exon 30 amino acid sequences from 8 diverse species shows that the exon is completely conserved in horses, humans, mice, dogs and cattle and contains only a few changes in the possum, chicken, and zebrafish (Figure S3). As LFS affected foals do not have an immunodeficiency consistent with *RAB27A* mutations, and the genomic region containing this gene was not inherited as predicted by our recessive model, it is doubtful that this gene plays a role in Lavender Foal Syndrome.

The newly discovered deletion in exon 30 of *MYO5A* leads to a frame shift and premature termination of transcription. Loss of the 379 amino acids at the C-terminus of the protein, which encode a portion of the secretory vesicle-specific binding domains of the globular tail, would likely impair binding of myosin Va to those cargo organelles bearing the appropriate receptors [18]. Although this truncation leaves intact the melanocyte specific alternative exon, exon F, it has been previously shown that binding function is nonetheless destroyed without the cooperative action of downstream motifs [19]. Additionally, the quantity of *MYO5A* protein may be significantly reduced, as is often observed in experimentally truncated constructs of this gene [19]. The resulting loss of vesicle traffic could easily interfere with the normal function of melanocytes and neurons. The neurologic deficits exhibited by LFS affected foals are relatively more severe than the symptoms reported in human cases of Griscelli Syndrome, which are most often due to changes in a single amino acid rather than loss of a significant portion of the transcript [16]. However, in the mouse a broad spectrum of phenotypes are observed, owing to the variety of causative mutations available for study.

There is some speculation that a mild, survivable epileptic condition of young foals may represent a non-lethal phenotype of LFS carriers [2] as the two conditions are often seen in the same pedigrees. However, this association has not been scientifically validated and samples from horses diagnosed with this condition were not available for study at this time. Based on comparative phenotypes in the mouse this is a plausible scenario. Several *MYO5A* alleles in the mouse, most notably mutations of the globular tail region like *d-n* and *d-n2J*, exhibit neurological and behavioral defects in juvenile homozygotes [20]. These deficits improve with age and are often survivable, as has been described in the rumored condition of the horse. Discovery of the mutation responsible for LFS will enable future studies to evaluate association of this allele with juvenile neurological dysfunction.

Our results suggest the population frequency of carriers of this deletion is 10.3% in the Egyptian Arabian. It is possible that this may be an over estimation of carriers, as owners who suspect they have LFS carrying horses may have been more motivated to participate in the study. However it is equally as likely that this figure is an underestimation as there is social stigma associated with producing LFS foals, thus motivating breeders to hide the carrier status of their breeding stock. Despite strict policies regarding the confidential nature of identifying information in research projects, this still influences some breeders to avoid association with Lavender Foal Syndrome research out of fear of being rumored to own carrier horses. Notably, three of the six carriers identified were reported to be breeding stallions. Data from the Egyptian Arabian horse registry indicates that approximately 850 young horses are registered each year (personal communication, Beth Minnich, Pyramid Society). Given our estimate of the number of carriers in the population we expect that around nine LFS foals would be born in the US each year. This is a small number; however rumors of carrier status can very quickly negatively impact the breeding career of high-priced stallions and lead to large economic losses. This estimate also assumes mating at random. In the case of the Egyptian Arabian horse this is not a realistic assumption given the commonplace use of inbreeding and line-breeding in the industry. The allele frequency for LFS of 5.2% is not unlike the frequencies of other heritable diseases in various breeds of horse [21], [22].

We identified a conserved block of 1.6 Mb in common in the four LFS bearing haplotypes. This is somewhat smaller than would be expected considering the average rate of decay of LD across just the six to eight generations that separate these four haplotypes. Indeed, upon further research of the pedigrees from carriers identified during screening for the LFS allele in our sample of 107 Arabian horses, we identified carriers who did not possess this candidate founder in their pedigree. Therefore it is likely the true founder of this mutation occurred far earlier. The appearance of a more recent common ancestor is not surprising given the prolific use of popular sires and the prevalence of inbreeding in this population.

Prevention of the economic and emotional losses associated with lethal conditions in foals, included those affected with LFS is a high priority among Arabian breeders. The market for Egyptian Arabian horses particularly values certain popular bloodlines. This leads to close breeding as owners seek to increase the percentage of this ancestry in their foal crop. This breeding strategy thus increases the need for vigilant prevention of recessive genetic disorders. The test developed here will be a pivotal tool for breeders seeking to breed within lines segregating for LFS, yet minimize or eliminate the production of affected foals.

Widespread application of the EquineSNP50 chip in genetic research is just beginning. In the case of Lavender Foal Syndrome, the limited availability of samples had impeded the progress of research using existing mapping tools for many years. Although whole genome association using large numbers of SNP markers is heralded as a tool for complex, polygenic traits, here we have shown that it can be very successfully applied to a simple trait in a small number of individuals. This work is the first use of the EquineSNP50 genotyping chip to successfully identify a causative mutation. While whole genome association is often the tool of choice for mapping complex traits and QTLs, we have demonstrated that it can also be a much anticipated solution for simple traits that face additional challenges in phenotyping and/or sample number. Testing for the LFS allele will be a valuable aid to breeders seeking to avoid losing foals while still using many of the popular lines that may carry Lavender Foal Syndrome. As the Arabian horse was used to develop many of the modern light horse breeds it is possible that the LFS allele is present in these breeds as well. In future work we will test additional sub-types of Arabian, as well as a variety of light horse breeds to better assess the population frequency in these groups. It is possible that LFS segregates in these groups at a low frequency without detection, as it is easy to confuse with other neonatal disorders of the foal.

## Materials and Methods

### Ethics Statement

Procedures in living animals were limited to the collection of blood by jugular venipuncture or hairs pulled from the mane or tail. Both procedures were conducted according to standard veterinary protocol and inflict minimal, if any pain. All samples were voluntarily submitted by horse owners and/or attending veterinarians to the Antczak or Brooks laboratories according to protocols approved by the Cornell Institutional Animal Care and Use Committee protocol #1986-0216.

### Horses

Six initial samples from affected foals plus one foal obtained mid-way through the study, their 31 relatives, as well as 114 individual horses from the general Arabian horse population were available for study. The diagnosis of Lavender Foal Syndrome was made by the attending veterinarian and was consistent with the previously published case reports [2], [3]. Population samples were voluntarily submitted by horse owners from across the US. As multiple samples received from a single owner often included closely related individuals, these horses were selected so as no horse included in the study was related to any other within a single generation. Although the identity and pedigree of study horses were made available, those data are not provided here to protect confidentiality. Each of the six affected horses had unique parents, although they were often related farther back in the pedigree (Figure S1). Samples were coded numerically during use to protect the anonymity of participating farms and owners.

### Sample Collection and Experimental Strategy

Although Lavender Foal Syndrome is widely-known among breeders of Arabian horses, the number of documented cases available for study and genetic analysis is very low. The six affected foals and their 30 relatives used for SNP genotyping in this study were collected over a 9 year period from Arabian breeders in various locations in the US. The average SNP density in the horse has been estimated at 1 per 2,000 bp [9]. It has been proposed that 100,000 SNPs should be sufficient for genome wide association mapping in the horse, given the moderate level of linkage disequilibrium both within and across breeds [9]. Due to the small number of available affected horses and the smaller than optimal horse SNP chip (only 56K SNPs), we decided to employ a modified family study using all of the available affected foals and their closest relatives, plus extensive pedigrees information available from the Arabian Horse DataSource (Arabian Horse Association, Aurora CO).

### DNA Extraction

Genomic DNA was isolated from fresh or frozen tissue or peripheral blood lymphocytes using the DNeasy Blood and Tissue kit (Qiagen Inc., Valencia, CA) following the manufacturer's protocol. DNA was eluted, as well as diluted in, MilliQ (Millipore Corp., Billerica, MA) water before use in downstream applications. Hair lysates were prepared for PCR from hair bulbs as previously published [23].

### Pedigree Analysis

The Lineage v.1.06 pedigree analysis program (Personal Communication, John Pollack, Animal Breeding Group Cornell University) was used to construct a pedigree and calculate F_i_ statistics as well as the coancestry coefficient for the 36 horses submitted for genotyping (Figure S1). For Figure S1 the “prune” option was used to hide individuals with fewer than two offspring as well as those with no known ancestors in order to simplify the pedigree for easier viewing.

### EquineSNP50 Genotyping and Analysis

We selected six affected foals, seven of their parents (all those from which samples were available) and 23 close relatives from amongst banked samples held at the Antczak laboratory. Genotyping on the EquineSNP50 chip was performed by the Genotyping Shared Resource at the Mayo Clinic, (Rochester, MN) using 75 µL of approximately 75 ng/µL genomic DNA. Across the 36 samples the genotyping call rate averaged 98% with a minor allele frequency of 0.47, on average. Genotypes were filtered to remove SNPs with a MAF <0.05 and missingness >0.5 using the Plink Whole Genome Analysis Toolset [24]. A Fisher's exact 3×2 test for a significant genotypic association between each SNP and the affected status was performed using the R statistical package v2.8.1 [25]. Statistical results were visualized and LD plots generated using Haploview [26] or the JMP v7.0 software package (SAS Institute Inc., Cary, NC). 281 SNPs from the significantly associated region were phased in to haplotypes using the Phase v2.1.1 [27]. Genome wide LD was estimated using the r^2^ statistic in the Plink Whole Genome Analysis Toolset under the following filters: minor allele frequency <0.05 and deviation from Hardy Weinburg Equilibrium p<0.0001. Ten individuals previously genotyped on the EquineSNP50 chip were chosen from the Arabian, Thoroughbred and Saddlebred breeds and compared to ten unrelated founder Egyptian Arabians typed for this study. Values were binned in groups of 5000 and average r^2^ and inter SNP distance graphed using Excel 2007 (Microsoft Corp., New York, NY).

### *MYO5A* Sequencing

As no full length mRNA sequence is currently available for *MYO5A* in the horse, exons were identified based on homology to the human mRNA sequence (NM_000259) aligned in the UCSC Genome Browser [28]. This human transcript, comprising 12,238 nt of mRNA (spanning 114 kb of genomic sequence) encoding 1855 amino acids, is 97.9% identical to the homologous equine sequence. Primers spanning these 39 exons were designed based on the EquCab2.0 genomic sequence from the UCSC Genome Browser using the Primer3 software [29] and purchased from Integrated DNA Technologies (Coralville, IA). These primers and their optimal annealing conditions are listed in Table S1. PCR products were submitted to the Cornell Core Life Sciences Laboratories Center for sequencing using standard ABI chemistry on a 3730 DNA Analyzer (Applied Biosystems Inc., Foster City, CA). All sequences were submitted to Genbank under the following accession numbers: GU183550 and GU183551. Sequences were aligned and screened for mutations using the Contig Express program in the Vector NTI Advance v10 suite (Invitrogen Corp., Carlsbad CA) or the CodonCode Aligner (CodonCode Corp., Dedham, MA)(Figure S3). The exon 30 sequence from an LFS horse was translated using Vector NTI Advance v10 and a multiple alignment constructed in Clustal X v.2 [30] using the following amino acid sequences from Genbank: horse XP_001918220.1, human EAW77447.1, mouse CAX15575.1, dog XP_535487.2, cow XP_615219.4, possum XP_001380677.1, chicken CAA77782.1, zebrafish AAI63575.1.

### PCR–RFLP Detection

25 ng of genomic DNA or 2 µL of hair lysate were amplified by PCR using the following primers: Myo5a.Exon30.RFLP.F 5′-CAG GGC CTT TGA GAA CTT TG-3′ and Myo5a.Exon30.R 5′-CAG CCA TGA AAG ATG GGT TT-3′. Reactions were assembled in a 10 µL total volume using FastStart Taq DNA Polymerase and included all reagents per the manufacturers recommended conditions (Roche Diagnostics, Indiananpolis, IN). Thermocycling on an Eppendorf Mastercycler Ep Gradient (Eppendorf Corp., Westbury, NY) was also according to the manufacturer's recommendations with an annealing temperature of 60°C and a total of 40 cycles for this primer pair. The restriction digest used 10 µL of PCR product, 1.5 units Fau I (New England Biolabs Inc. (NEB), Ipswitch, MA), 1x NEB Buffer 4 and enough MilliQ water to bring the reaction volume to 20 uL. Digests were incubated at 55°C for 1 hour. The resulting products were combined with loading buffer (Gel Loading Dye (6X), NEB) and separated alongside a size standard (100 bp DNA Ladder, NEB) by electrophoresis following standard conditions on a 3% agarose gel (Omnipur Agarose, EMD Chemicals Inc, Gibbstown, NJ). Agarose gels were stained (SYBRsafe DNA gel stain (10,000X) concentrate, Invitrogen Molecular Probes, Eugene, OR) and visualized under UV illumination (FluroChem HD2, Alpha Innotec Corp., San Leandro CA).

## Supporting Information

Figure S1 Pedigree of horses used in this study. Red indicates affected, blue indicates carriers, and green highlights horses chosen for genotyping.
(0.10 MB TIF)

Figure S2 Average genome-wide LD in four horse breeds. The Egyptian (sub-group of the Arabian) and the Thoroughbred, both with a long history of a closed stud book, have relatively long LD in contrast to the Arabian and Saddlebred breeds.
(0.12 MB TIF)

Figure S3 Multiple alignment of *MYO5A* exon 30 amino acid sequences from eight diverse species and an LFS horse demonstrating the high level of conservation in this region of the gene. The first amino acid changed by the g.138235715del is marked with a yellow arrow. A star denotes completely conserved amino acids.
(1.12 MB TIF)

Figure S4 Detection of the Lavender Foal Syndrome associated deletion by PCR-RFLP. Digestion products were visualized by electrophoresis on a 3% gel under UV illumination following staining with SYBR green. A lane of size standard is on the far left followed by (A) a negative control, (B) undigested PCR and the digestion products in lanes, (C) from a normal horse, (D) a carrier, and finally (E) an affected foal.
(0.35 MB TIF)

Table S1 Primers sequences used to amplify 39 exons of *MYO5A*.
(0.14 MB RTF)
