# Supplementary figures and images for: Whole-Genome SNP Association in the Horse: Identification of a Deletion in Myosin Va Responsible for Lavender Foal Syndrome
Source: PLoS Genet. 2010 Apr 15;6(4):e1000909. doi: 10.1371/journal.pgen.1000909 (PMC2855325; doi:10.1371/journal.pgen.1000909)

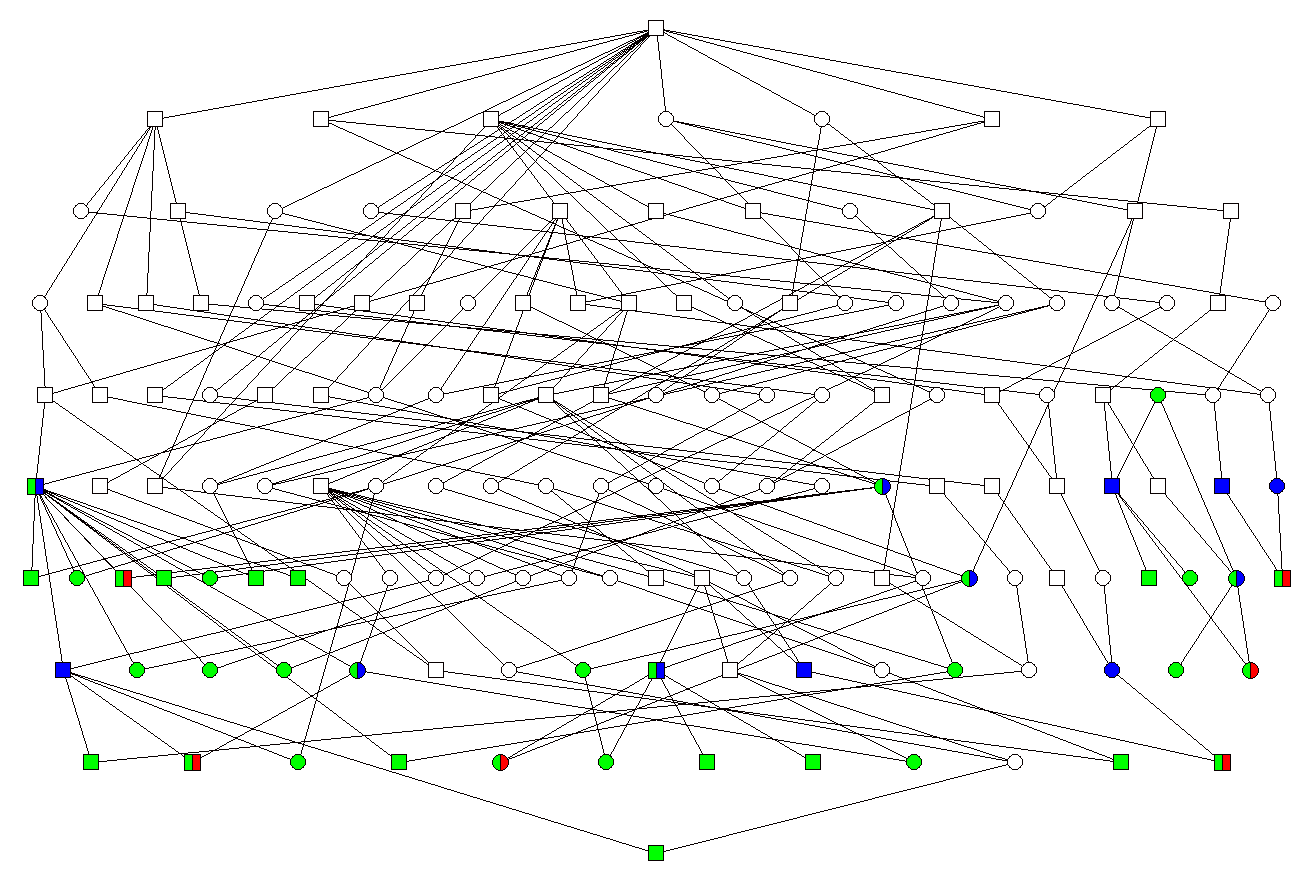

Supplement: Figure S1 — Pedigree of horses used in this study. Red indicates affected, blue indicates carriers, and green highlights horses chosen for genotyping. (0.10 MB TIF) [file pgen.1000909.s001.tif]

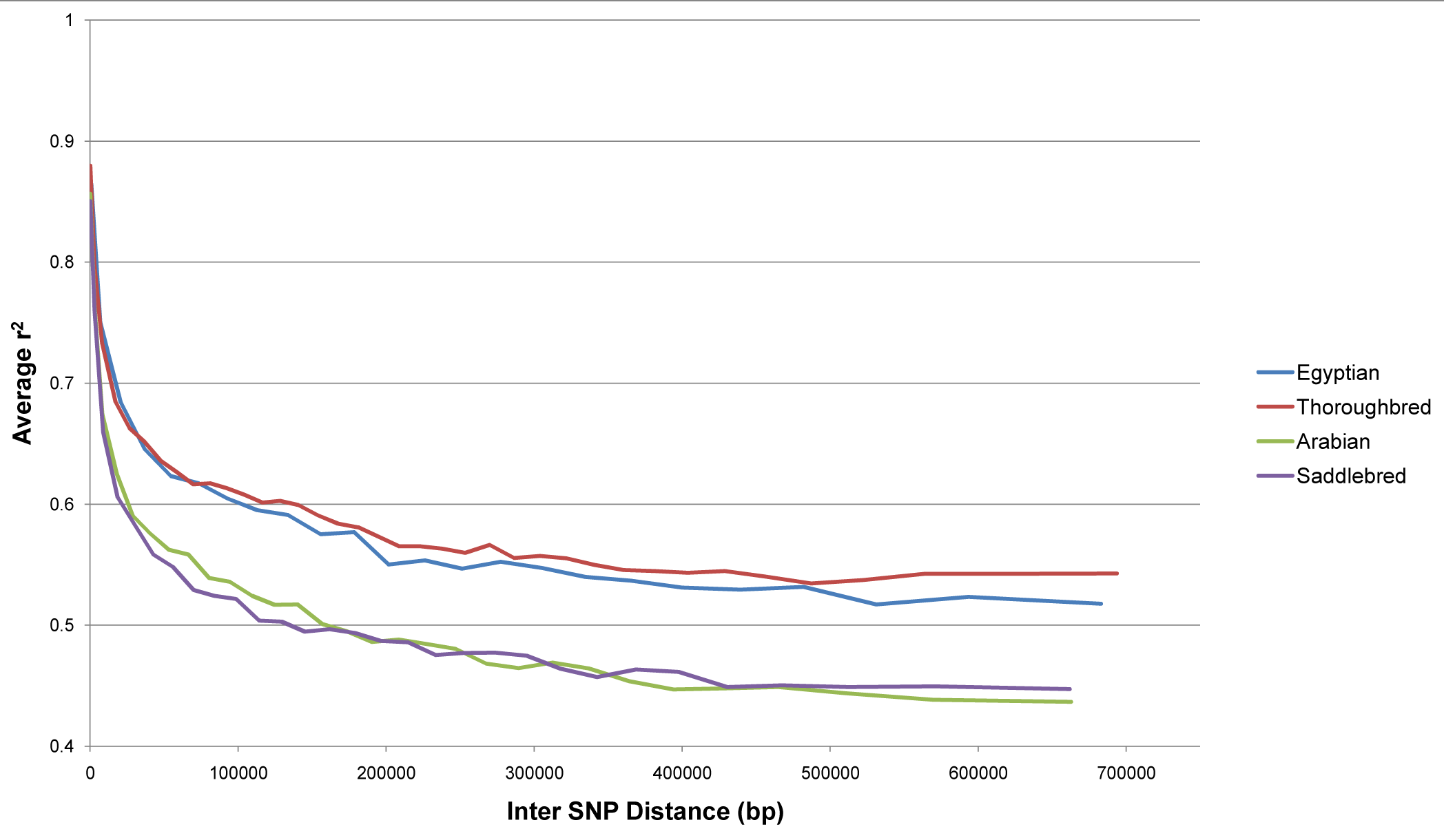

Supplement: Figure S2 — Average genome-wide LD in four horse breeds. The Egyptian (sub-group of the Arabian) and the Thoroughbred, both with a long history of a closed stud book, have relatively long LD in contrast to the Arabian and Saddlebred breeds. (0.12 MB TIF) [file pgen.1000909.s002.tif]

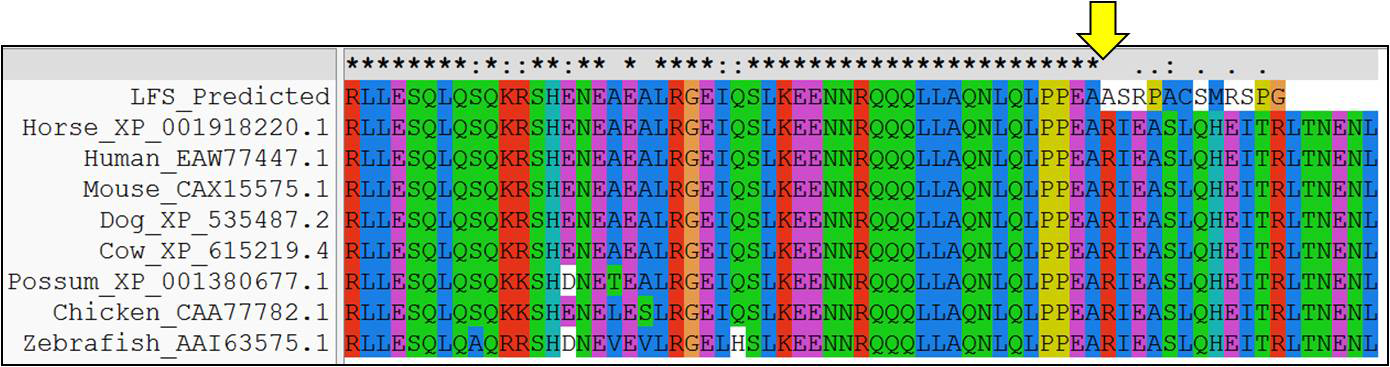

Supplement: Figure S3 — Multiple alignment of MYO5A exon 30 amino acid sequences from eight diverse species and an LFS horse demonstrating the high level of conservation in this region of the gene. The first amino acid changed by the g.138235715del is marked with a yellow arrow. A star denotes completely conserved amino acids. (1.12 MB TIF) [file pgen.1000909.s003.tif]

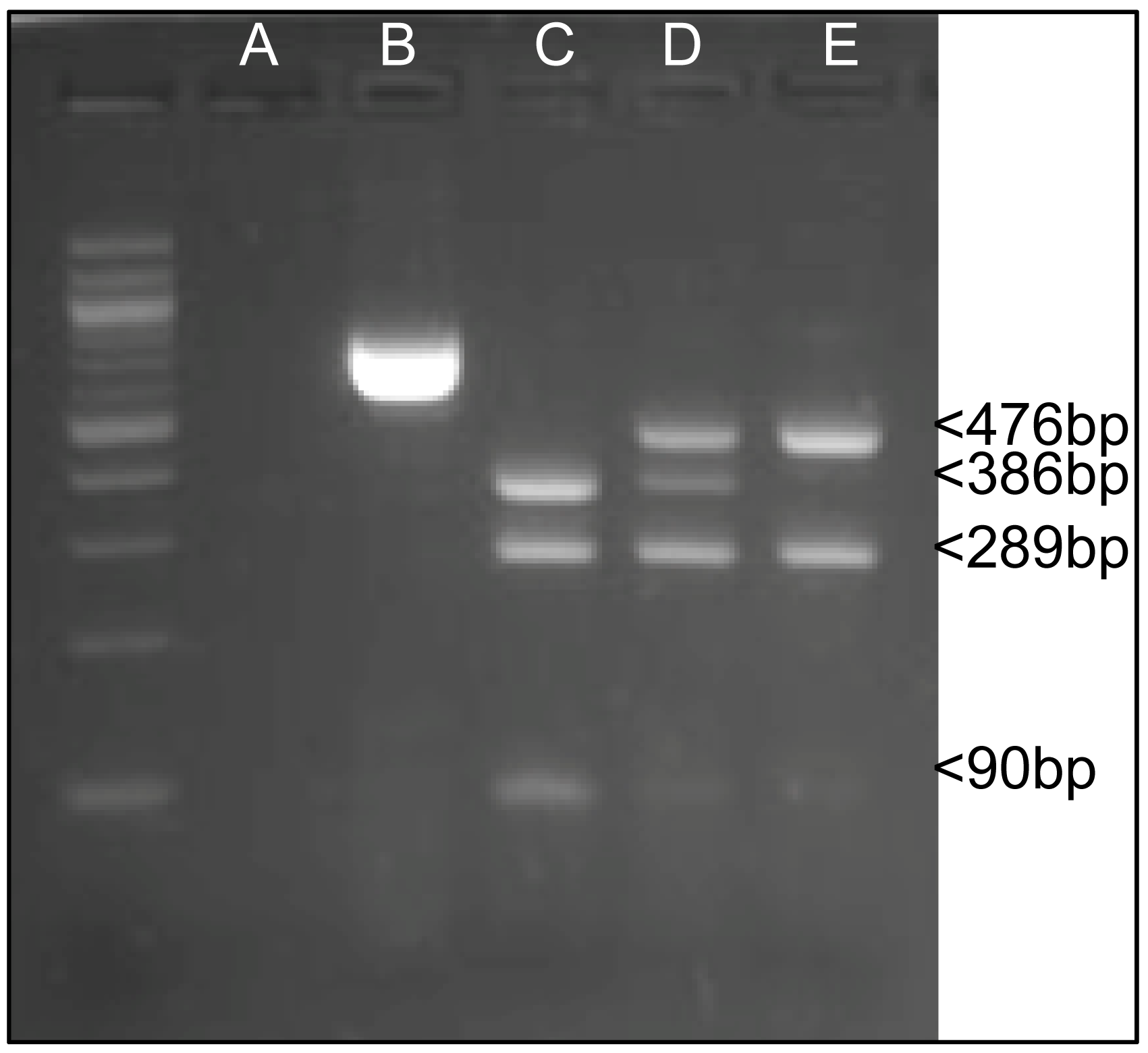

Supplement: Figure S4 — Detection of the Lavender Foal Syndrome associated deletion by PCR-RFLP. Digestion products were visualized by electrophoresis on a 3% gel under UV illumination following staining with SYBR green. A lane of size standard is on the far left followed by (A) a negative control, (B) undigested PCR and the digestion products in lanes, (C) from a normal horse, (D) a carrier, and finally (E) an affected foal. (0.35 MB TIF) [file pgen.1000909.s004.tif]
