# Supplementary material for: Whole-Genome SNP Association in the Horse: Identification of a Deletion in Myosin Va Responsible for Lavender Foal Syndrome
Source: PLoS Genet. 2010 Apr 15;6(4):e1000909. doi: 10.1371/journal.pgen.1000909 (PMC2855325; doi:10.1371/journal.pgen.1000909)
Supplement: Table S1 — Primers sequences used to amplify 39 exons of MYO5A. (0.14 MB RTF) [file pgen.1000909.s005.rtf]

Exon	Forward Name	Forward Location	Sequence 5'-3'	Reverse Name	Reverse Location	Sequence 5'-3'	Annealing Temp, oC	
1	Myo5a.Exon1.F	138142209	ACA GCG TCG TGA TGT GAC TT	Myo5a.Exon1.R	138142735	GAG TTT GCC ATT TTG CCA TT	60	
2	Myo5a.Exon2.F	138145887	ACC AGT TTC TGT GGG TTT GG	Myo5a.Exon2.R	138146393	GCG TGC CGA ACT TAA TCA CT	60	
3	Myo5a.Exon3.F	138148327	GAA TGA GGC AAA GAC TGT GAT G	Myo5a.Exon3.R	138148893	TCT GGT CTG CCA CCC ATT AG	60	
4	Myo5a.Exon4.F	138160959	AAA CTC ACC CTG CCT CAC TG	Myo5a.Exon4.R	138161486	GCC AGC TGT TTA GAG GCA AC	60	
5	Myo5a.Exon5.F	138165302	TGC TGG TAT GAA TGG GTG TG	Myo5a.Exon5.R	138165881	TCC TCA GCC TCT GAC AGT CC	60	
6	Myo5a.Exon6.F	138167607	GCT TCT CCA CAA AGG CAA TG	Myo5a.Exon6.R	138168165	GCC ACT CAC ACC TTC TCC AT	58	
7	Myo5a.Exon7.F	138168307	TAT GTG ATG GAG GCC TGT GA	Myo5a.Exon7.R	138168874	TTT GTT CCA GTT GCT TAC CAA G	58	
8	Myo5a.Exon8.F	138171206	CTC CAC AGC TCA TGC TCT CA	Myo5a.Exon8.R	138171804	TCA CCC ATC CAA CTT TCA CA	58	
9	Myo5a.Exon9.F	138176691	TGG TGG GTT TTG TTT GTT ACC	Myo5a.Exon9.R	138177221	GGG TAC CCA GAG AAG GGA AA	58	
10	Myo5a.Exon10.F	138177376	CAA GAG CAA TGG CTT TGT CA	Myo5a.Exon10.R	138177901	CCT GCT TCC CTC ACT CAC TT	58	
11	Myo5a.Exon11.F	138184287	TCC CAT GAG ATT GTT GTT GC	Myo5a.Exon11.R	138184788	GAA TCA GCT GAA ATG AGA AGG AA	60	
12	Myo5a.Exon12.F	138186095	CCA TGG GAA GGT CTG TCA TT	Myo5a.Exon12.R	138186656	CGG TTT TTG GAG TTT CTT GG	60	
13	Myo5a.Exon 13.F	138187544	CGT GCT TCT TAC CGA GAA GG	Myo5a.Exon13.R	138188045	CTG TGC CTC AGT TCC CTC AT	61	
14	Myo5a.Exon14.F	138192630	CTG GCA TGT GAC TCT GAA GG	Myo5a.Exon14.R	138193153	AAA CAA AGG TGG CAG TGG TC	61	
15	Myo5a.Exon15.F	138193984	GAG GGT GGG TCA GGT TCA TA	Myo5a.Exon15.R	138194487	CCA ACA AAA GCC CAG AAA GA	61	
16+17	Myo5a.Exon16+17.F	138195951	TGA TTG TCC CTC CTG ACT CC	Myo5a.Exon16+17.R	138196543	CCA CAC ACC CCC AAA TAA TC	61	
18	Myo5a.Exon18.F	138198401	ACC AAG TTT TTG GCC TCA GA	Myo5a.Exon18.R	138198991	TCA GGT AGG GTG ACC ACT CA	60	
19	Myo5a.Exon19.F	138199481	TAC CCT GGG ACA GTG AGG AC	Myo5a.Exon19.R	138200069	CCC TAT GGC TCC TAC ATC CA	61	
20	Myo5a.Exon20.F	138203498	GCC TAG TCC AGG GTC TGA CA	Myo5a.Exon20.R	138204020	AGG CTT GGA GTG CTG GTA AA	60	
21	Myo5a.Exon21.F	138204596	TTG TGC CTC TTC TCT TTT CTT TG	Myo5a.Exon21.R	138205117	CTA CTT GGG CCA GGT CAA GA	61	
22	Myo5a.Exon22.F	138207639	CTG CAG CCA TCC CAG TTT AT	Myo5a.Exon22.R	138208171	AGT TTG GTG GCA AAG AAT GG	61	
23	Myo5a.Exon23.F	138209968	AAA ACC CCT TGC AGA GAA GC	Myo5a.Exon23.R	138210565	CCA GCG GAG GAA ATA CTC AA	61	
24	Myo5a.Exon24.F	138214127	TCA TTC CTG TGC TTC ACT CG	Myo5a.Exon24.R	138214636	ACT CGG CAA TGG AAT TGT TC	61	
25+26	Myo5a.Exon25+26.F	138220984	TGT TGG GCC GTT TAT CAT TT	Myo5a.Exon25+26.R	138221566	CCC TTG CCA AAA CAA GAC AT	61	
27	Myo5a.Exon27.F	138225186	CTT GCT CAA CTC CCC TTG AC	Myo5a.Exon27.R	138225769	TGG CAT TGG CAA CAG TTT TA	61	
28	Myo5a.Exon28.F	138230184	ACA GGG CAG TGG TAC TCT GG	Myo5a.Exon28.R	138230709	TTG GAT TCC CAG TGA AGG TC	61	
29	Myo5a.Exon29.F	138233269	GCC ACA TGA AGA CAG TAG CTG A	Myo5a.Exon29.R	138233833	AAA GTC AGG ACG CCA CTG TT	61	
30	Myo5a.Exon30.F	138235546	CCT ACC TGT GTG GCT TCT CC	Myo5a.Exon30.R	138236107	CAG CCA TGA AAG ATG GGT TT	62	
31	Myo5a.Exon31.F	138239588	ACC CAC ATG GAG CTT ACT GG	Myo5a.Exon31.R	138240115	ATT TTC CCC AAC CTT GCA GT	61	
32	Myo5a.Exon32.F	138244844	TTG ACA GTA GCC AAG CAC CA	Myo5a.Exon32.R	138245368	CAA GTG TCC CTG CAG TTT GA	62	
33	Myo5a.Exon33.F	138246929	TCA GTT TGA CAT GCC TGA GC	Myo5a.Exon33.R	138247507	ACG GAA GGT GTC GGT TAA GA	62	
34	Myo5a.Exon34.F	138249171	TAA CCT GTG GCT AGG CGA AT	Myo5a.Exon34.R	138249681	CCC TGA ACT CAG GTT TCC AA	62	
35	Myo5a.Exon35.F	138251018	TGG CAT TTA TGT GTG GGA TG	Myo5a.Exon35.R	138251565	AGA GCG AGG CAT ACA GCA CT	62	
36	Myo5a.Exon36.F	138253274	ACG TGA CCT GAT GAG CAG TG	Myo5a.Exon36.R	138253827	CAC AGG TGT TGG TGT TTG CT	62	
37	Myo5a.Exon37.F	138255228	GGA CAA AGC CAC ACA CAA AA	Myo5a.Exon37.R	138255784	ACA AGC TCC GTG GTA TGT CC	62	
38	Myo5a.Exon38.F	138256360	GGG GGC ACT TGT ACT TTT CA	Myo5a.Exon38.R	138256956	TCT GCC AAA AAT TCT ACC AAA AA	62	
39	Myo5a.Exon39.F	138256934	TTT TTG GTA GAA TTT TTG GCA GA	Myo5a.Exon39.R	138257440	CCT TCC TTT CGT CAT TCA CC	62	
